# Polypharmacy, drug–drug interactions, and potentially inappropriate medications among older adults: a cross-sectional study in Northeast Ethiopia

**DOI:** 10.3389/fpubh.2025.1525079

**Published:** 2025-04-17

**Authors:** Bedilu Linger Endalifer, Mekuanint Terefe Kassa, Yenesew Wudu Ejigu, Abyou Seyfu Ambaye

**Affiliations:** ^1^Departement of Pharmacy, Asrat Woldeyes Health Science Campus, Debre Berhan University, Debre Berhan, Ethiopia; ^2^School of Medicine, Asrat Woldeyes Health Science Campus, Debre Berhan University, Debre Berhan, Ethiopia; ^3^School of Pharmacy, College of Medicine and Health Science, Wollo University, Dessie, Ethiopia

**Keywords:** polypharmacy, drug–drug interaction, potential inappropriate medication, Beer’s Criteria, START/STOP criteria, comorbidity

## Abstract

**Background:**

The global older adult population is expected to increase from 524 million in 2010 to 1.5 billion by 2050, mainly in developing countries. Age-related diseases, comorbidities, and polypharmacy make appropriate prescribing crucial. This study aimed to assess the prevalence of polypharmacy, drug–drug interaction, and potentially inappropriate medication use and its factors in an Ethiopian hospital.

**Methods:**

A facility-based cross-sectional study on 236 patients aged 65 and above at Dessie Comprehensive Specialized Hospital (Jan 2022–Apr 2023) used the 2023 Beers Criteria and START/STOP V.3 to identify potentially inappropriate medications. Polypharmacy and potential drug–drug interactions were assessed using Micromedex®, with descriptive statistics and binary logistic regression performed in SPSS version 26.

**Result:**

Of the 236 patients in this study, 94 (39.8, 95% CI: 35.7–44.5%) were prescribed at least one potentially inappropriate medication per the STOPP/START criteria, with 81 (34.3%) identified by STOPP and 13 (5.5%) by START. According to the Beers Criteria, 108 patients (45.7, 95% CI: 40.1–51.0%) received at least one potentially inappropriate medication. Polypharmacy was observed in 80 patients (33.9, 95% CI: 29.1–38.5%), and potential drug–drug interactions were identified in 111 patients (47.0%). Being female (AOR: 2.93), age ≥75 (AOR: 1.52), and polypharmacy (AOR: 3.20) were linked to potentially inappropriate medication use per Beers Criteria. Age 70–74 (AOR: 2.30) and polypharmacy (AOR: 3.10) were also associated per STOPP/START criteria.

**Conclusion:**

Polypharmacy, drug–drug interactions, and potentially inappropriate medications are common among older Ethiopian patients, with age, sex, and polypharmacy as contributing factors. Future studies are needed to assess the health and economic impacts of potentially inappropriate medications use.

## Background

The global population of individuals aged 65 and above is projected to increase significantly, from 524 million in 2010 to an estimated 1.5 billion by 2050, with the majority of this growth occurring in developing countries (1). Drug therapy for the older adult requires special consideration due to age-related changes in pharmacokinetics and drug sensitivity (2). Older adults are at higher risk for age-related comorbidities, often necessitating multiple medications, which can lead to inappropriate and potentially harmful prescribing practices. Inappropriate prescriptions remain a significant health concern in the older adult population (3).

Older adults face a high risk of inappropriate medication use due to their complex medical conditions and the use of multiple medications (4). Certain drugs pose an increased risk of adverse drug events in this population and are thus classified as potentially inappropriate medications (PIMs) for older adults (5). Medication appropriateness is crucial in geriatrics due to their heightened vulnerability to medication issues. Screening and intervention strategies using accessible tools can be integrated into clinical workflows and, when possible, electronic medical records to improve medication safety (4). Among nursing home residents, approximately 50% are exposed to PIMs, with data suggesting an increasing prevalence over time (6).

Inappropriate prescribing is significantly associated with the number of medications prescribed to older adult patients (7). The prevalence of PIM varies across countries; for instance, studies report PIM exposure rates of 52.5% in Saudi Arabia (5), 11% in the USA (8), 66% in India (9), and 88% in Germany (10) among older adult patients. A retrospective study in the USA on cardiovascular patients observed a high prevalence of PIM use in the geriatric population (11). Similarly, a retrospective study in northwest Ethiopia involving 1,252 patients found that 347 (27.7%) received at least one PIM, with immediate-release nifedipine (53.9%) being the most inappropriately prescribed, followed by diclofenac (22.2%), ibuprofen (7.8%), and indomethacin (5.2%) (12). Additionally, an observational study in India indicated a high prevalence of potentially inappropriate prescribing and adverse drug reactions among hospitalized older adults (13).

A significant association was found between PIMs and adverse outcomes, including increased hospitalizations, emphasizing the need for interventional strategies to prevent PIM use, especially in patients with multiple chronic conditions (14). The use of PIMs among the geriatric population is linked to an increased risk of unplanned hospitalizations, especially among patients undergoing polypharmacy, warranting caution in PIM prescriptions for older adults (15). PIMs pose a substantial risk of adverse drug events in older adult patients (5), and their use is a significant concern due to the higher demand for emergency care resources annually (16). Reducing PIM utilization can decrease adverse drug reactions, lower treatment costs, enhance medication adherence, and reduce hospitalization risk in older patients (7, 17).

In Ethiopia, the absence of specific guidelines for geriatric medication management underscores the importance of this study. Findings may inform the development of guidelines to improve medication appropriateness and reduce PIMs among older adults. Additionally, this study can aid institutions in updating formularies and incorporating safer alternatives, ultimately optimizing drug therapy for older adult patients.

## Methods

### Study design and study population

A facility-based cross-sectional study was conducted among patients aged 65 and above who attended the medical referral clinic at Dessie Comprehensive Specialized Hospital from January 1, 2022, to April 30, 2023. In these clinics, patients typically have a maximum follow-up period of 4 months; hence, data collection was conducted over 4 months. Patients with incomplete medical or medication records such as missing dosage or frequency information and those on follow-up without any prescribed medications were excluded from the study.

### Sample size determination

The sample size was calculated using a single proportion formula, based on a previous study that reported a 28.6% prevalence of PIMs (12), with a 5% margin of error and a 95% confidence level. During the study period, 675 older adult patients visited Dessie Comprehensive Specialized Hospital. After applying a finite population correction, a final sample of 236 patients was determined. Participants were selected through simple random sampling.


$$n=\frac{\left(0.286*0.714\right)1.96^{2}}{0.05*0,05}=314$$

By adjusting this number $\frac{314*675\;}{314+675}$ = 214 by adding 10% 236.

### Data collection, procedure, and instrument

To enhance patient safety, the STOPP/START criteria and AGS Beers Criteria provide updated guidelines for PIMs in older adults. These guidelines highlight medications that may increase the risk of adverse drug reactions and offer safer alternatives or recommendations for cautious use. The STOPP/START version 3 criteria serve as tools to improve medication management in this population. The STOPP criteria identify potentially inappropriate medications to be avoided, while the START criteria emphasize essential medications that may be under-prescribed. This version focuses on reducing medication-related risks by optimizing treatment and minimizing unnecessary polypharmacy. A data abstraction tool was developed utilizing the 2023 AGS Beers Criteria (18), the STOPP/START version 3 (19), and previously published articles (20, 21). This tool was employed to extract essential information, including patient demographics, medical conditions, medication-related details, and relevant investigative data, using a pre-designed data abstraction format.

### Variables of the study

The analysis considered several independent variables, including demographic factors (age in years and gender), the number of comorbidities, specific comorbid conditions, and the total number of prescribed medications. Chronic disease conditions were classified: as cardiovascular, endocrine, respiratory, neurological, hematologic, and ophthalmic disorders, which were treated as additional independent variables. The primary outcome of interest was the incidence of PIMs, while secondary outcomes included instances of polypharmacy and drug–drug interactions. While no universally accepted definition of polypharmacy exists, this study defined polypharmacy as the concurrent prescription of five or more medications (22). Potential drug–drug interactions (pDDIs) were evaluated using the online computerized verification system available through Micromedex®. This tool classifies pDDIs by severity: mild or minor drug interactions are deemed to have minimal clinical significance and are unlikely to produce relevant clinical effects. In contrast, moderate interactions may have clinical implications and should be approached with caution, necessitating close monitoring, as they could exacerbate the patient’s condition or require therapy adjustments. Severe drug interactions are clinically significant, potentially life-threatening, and may necessitate medical intervention to mitigate or prevent serious adverse effects (23).

### Data entry and analysis

Data were coded, cleaned, entered, and analyzed using SPSS version 26. Descriptive statistics, including mean, median, and percentages, were calculated, and results were presented in tables and charts. Binary logistic regression analysis was performed, with variables exhibiting a *p*-value of <0.05 considered statistically significant in the multivariate analysis. The odds ratio (OR) and 95% confidence interval were calculated for each variable to assess the strength of the associations.

## Results

### Socio-demographic and clinical characteristics of patients

In this study, the mean age of participants was 70.5 ± 5.9 years, with males representing 119 (50.4%) of the cohort. A substantial proportion, 169 (71.6%), of participants had comorbid conditions. A total of 743 medications were prescribed, resulting in an average of 3.2 ± 1.7 medications per patient. Notably, 66 (28%) of patients were prescribed only one medication, whereas 80 (33.9%) received five or more medications (Table 1).

**Table 1:** Demographic characteristics of study participants at Dessie Comprehensive Specialized Hospital, Ethiopia (*N* = 236).

Characteristics	Category	Frequency	Percent
Gender	Male	119	50.4
	Female	117	49.6
Age (mean ± Standard Deviation)	70.51 ± 5.892		
	65–69	124	52.6
	70–74	65	27.5
	> = 75	74	19.9
Comorbidity	Yes	169	71.6
	No	67	28.4
Number of comorbidities	2.67 ± 1.869		
	One	67	28.4
	Two	108	45.8
	Three and above	61	25.8
Number of drugs (mean ± Standard Deviation)	3.15 ± 1.66		
	1	66	28
	2–4	90	38.1
	> = 5	80	33.9

### Disease characteristics

Diabetes mellitus was the most prevalent condition, with a prevalence of 99 (42%), followed by hypertension at 70 (29.7%). Chronic kidney disease and chronic obstructive pulmonary disease were the least prevalent, affecting 5 (2.1%) and 4 (1.7%) of participants, respectively (Table 2).

**Table 2:** Disease characteristics of the study participants at Dessie Comprehensive Specialized Hospital, Ethiopia (*N* = 236).

Disease	Category	Frequency	percentage
Cardio vascular disease	Hypertension	70	29.7
	Heart Failure	60	25.4
	Previous Acute coronary syndrome	28	11.9
	Stroke	32	13.6
Endocrine disorder	Diabetes Mellitus	99	42
	Thyroid Disorder	41	17.4
Gastro intestinal Disorders	Peptic ulcer disease	28	11.9
	Gastroesophageal reflux disease	13	5.5
Renal disorder	Acute kidney disease	8	3.4
	Chronic kidney disease	5	2.1
Respiratory Disorder	Asthma	10	4.2
	Chronic Obstructive Pulmonary Disease	4	1.7
Neurologic Disorder	Parkinson’s Disease	9	3.8
	Dementia	8	3.4
Ophthalmic Disorder	Glaucoma	11	4.7
Hematology disorder	Anemia	8	3.4

### Potentially inappropriate medications based on STOPP/START criteria

According to the STOPP/START criteria, 94 participants (39.8, 95% CI: 35.7–44.5%) were prescribed at least one PIM, with 81 (34.3%) identified by STOPP criteria and 13 (5.5%) by START criteria. Among the STOPP-listed medications, long-acting sulfonylureas (Glibenclamide) were the most frequently prescribed, affecting 61 patients. From the START criteria, the most common omission was the failure to initiate cardio-selective beta-blockers in patients with potential added benefits, affecting 4 (1.7%) patients (Table 3).

**Table 3:** Potentially inappropriate medications based on STOPP/START criteria at Dessie Comprehensive Specialized Hospital, Ethiopia (*N* = 236).

STOPP criteria	Frequency
Glibenclamide prescribed for type 2 diabetes mellitus	61
Long-term corticosteroids (>3 months) as monotherapy for rheumatoid arthritis	5
Paracetamol at doses ≥ 3 g/24 h in patients with poor nutritional status	4
Tricyclic Antidepressants in patients with dementia and orthostatic hypotension	3
First-generation antihistamines as first-line treatment for allergy	3
Angiotensin-converting enzyme inhibitors in patients with hyperkaliemia	2
Duplicate drug class prescription for daily regular use	2
Nonsteroidal antiinflammatory drugs for eGFR < 50 mL/min/1.73m^2^	2
Proton pump inhibitor for uncomplicated peptic ulcer disease for > 8 weeks	2
Digoxin for heart failure with normal systolic ventricular function	1
Levothyroxine in subclinical hypothyroidism	1
Long-term aspirin at doses greater than 100 mg per day	1
Long-term systemic NSAIDs with a known history of coronary disease	1
START criteria	
Cardio-selective beta-blocker for stable heart failure with reduced ejection fraction.	4
Disease-modifying anti-rheumatic drug with chronic, active, and disabling rheumatoid arthritis.	3
Sacubitril/valsartan in heart failure with reduced ejection fraction causing persistent heart failure symptoms	3
Proton pump inhibitor with initiation of low-dose aspirin and previous history of peptic ulcer or reflux esophagitis.	2
Mineralocorticoid receptor antagonist (spironolactone, eplerenone) in heart failure without severe renal function impairment, i.e., eGFR > 30 mL/min/m2	1

NSAIDs: Nonsteroidal anti-inflammatory drugs, GFR: Glomerular filtration rate.

### Potentially inappropriate medications based on AGS Beers Criteria

A total of 108 older patients (45.7%; 95% CI: 40.1–51.0%) were prescribed at least one PIM according to the Beers Criteria. The medication class deemed potentially inappropriate had the highest occurrence, affecting 81 patients, while medications requiring dosage adjustment based on renal function but not adjusted had the lowest prevalence, involving 5 patients (Table 4).

**Table 4:** Potentially inappropriate medications based on Beers Criteria Dessie Comprehensive Specialized Hospital, Ethiopia (*N* = 236).

American Geriatrics Society Beers Criteria	Frequency
Medications considered potentially inappropriate	81
Medications potentially inappropriate in patients with certain diseases or syndromes	17
Potentially inappropriate drug–drug interactions	14
Medications to be used with caution	8
Medications whose dosages should be adjusted based on renal function	5

### Polypharmacy and drug–drug interaction

In this study, 33.9% of patients (95% CI: 29.1–38.5%) experienced polypharmacy, defined as the use of five or more medications. All polypharmacy patients had at least two comorbidities. Potential drug–drug interactions were found in 47.0% of patients (111), with an average of 2.36 ± 2.10 interactions per patient, totaling 551 interactions. The majority were moderate (47.9%, 264), followed by major (35.2%, 194) and minor (29.8%, 93) interactions (Table 5).

**Table 5:** Major drug–drug interaction at Dessie Comprehensive Specialized Hospital, Ethiopia (*N* = 236).

Drug-drug interaction	Frequency	Possible outcome
ASA + Glibenclamide	33	May result in increased risk of hypoglycemia.
ASA + Metformin	25	Result in increased risk of hypoglycemia.
ASA + Furosemide	19	Reduce diuretic effectiveness and nephrotoxicity.
ASA + HCT	17	Decrease diuretic effectiveness and kidney toxicity
ASA + Warfarin	16	may increase the risk of bleeding
ASA + Spironolactone	15	Reduce diuretic effect, hyperkalemia, kidney toxicity
Enalapril + Spironolactone	13	Hyperkalemia.
ASA + Diclofenac	7	Increase risk of bleeding and cardiovascular events.
Ciprofloxacin + Warfarin	5	Increased risk of bleeding.
Diclofenac + furosemide	5	Reduce diuretic effect and cause kidney toxicity
Ciprofloxacin + Metformin	4	Hypo- or hyperglycemia.
Digoxin + Nifedipine	4	Causes digoxin toxicity
Digoxin + spironolactone	4	Increased digoxin exposure
Atorvastatin + digoxin	3	Increased plasma concentrations of digoxin.
ASA + Indomethacin	3	Increased bleeding and cardiovascular risks
Diclofenac +HCT	3	Decreased diuretic effect and kidney toxicity.
Digoxin + Furosemide	2	Increased risk of digoxin toxicity
Digoxin + Metoprolol succinate	2	increased risk of bradycardia and digitalis toxicity
ASA + Digoxin	2	Increase digoxin levels and extend its half-life.
Furosemide + Indomethacin	2	Increased risk of renal failure and reduced antihypertensive and diuretic effectiveness.
Glibenclamide + Norfloxacin	2	Increased risk of hypoglycemia or hyperglycemia.
Indomethacin + Spironolactone	2	Increased serum potassium levels or acute renal failure
Metformin + Norfloxacin	2	Increased risk of hypoglycemia or hyperglycemia.
ASA + Clopidogrel	2	May result in an increased risk of bleeding.
Ciprofloxacin + Glibenclamide	2	Hypo- or hyperglycemia.

ASA, Acetylsalicylic acid; HCT, Hydrochlorothiazide.

### Factors associated with Beer’s PIM criteria

Sex, age, presence of comorbidity, and number of drugs were analyzed independently by bivariate and multivariate logistic regression for Beers and STOPP/ START PIMs. Female patients≥ 75 years old are 2.9 3 and 1.52 times more likely to have Beer’s PIMs. Polypharmacy prescription (≥5 drugs) increases the likelihood of Beer’s PIMs by 3.20 times as compared to the single drug. For STOPP/START criteria. Age of 70–74 patients 2.3 times and polypharmacy (≥5) drugs 3.20 times chance of having STOPP/ START PIMs list as compared to the age of 65–69 and only on one drug (Table 6).

**Table 6:** Association of contributing factors and PIMs in Dessie Comprehensive Specialized Hospital, Ethiopia (*N* = 236).

Variable	Category	PIM		PIM present	
		Yes	No	COR (75% CI)	AOR (95% CI)
AGS Beers PIMs					
Sex	Male	52	67	1.00	1.00
	Female	56	61	4.928 (3.53, 5.7)	2.93 (2.54, 3.60)*
Age	65–69	43	81	1.00	1.00
	70–74	30	35	0.53 (0.76, 2.80)	0.96 (0.51, 1.81)
	≥75	35	39	2.928 (2.539, 3.599)	1.52 (1.127, 2.329)*
Comorbidity	Present	85	84	3.08 (1.66, 3.55)	1.08 (0.46, 2.52)
	Absent	23	44	1.00	1.00
Number of drugs	1	15	51	1.00	1.00
	2–4	47	43	2.77 (3.87, 5.58)	0.57 (0.876,3.43)
	≥5	46	34	0.98 (0.25, 2.53)	3.20 (1.59, 6.69)*
STOPP/START PIMs					
Sex	Male	48	71	1.00	1.00
	Female	46	71	4.20 (0.18, 0.57)	3.02 (0.81, 2.07)
Age	65–69	36	88	1.00	1.00
	70–74	22	43	4.21 (2.40, 5.79)	2.30 (1.78, 4.59)*
	≥75	36	41	2.34 (0.97, 2.49)	3.10 (0.23, 0.89)
Comorbidity	Present	70	99	1.22 (0.36, 4.63)	4.04 (0.06, 0.93)
	Absent	24	43	1.00	1.00
Number of drugs	1	17	49	1.00	1.00
	2–4	31	59	1.204 (0.586, 1.693)	1.204 (0.586, 1.693)
	≥5	46	24	2.21 (2.66, 4.99)	3.10 (1.56, 3.43)*

*p-value is significant at <0.05: CI, confidence interval; AOR, adjusted odds ratio; COR, Crude odds ratio.

## Discussion

This study revealed a prevalence of 39.8% for STOPP/START criteria and 45.7% for Beers criteria PIMs, which aligns with findings from various countries worldwide, ranging from 44 to 52% (24–27). The slightly lower prevalence compared to other studies (9, 28–32) may be due to differences in study settings, study designs, and the criteria used to identify PIMs. Additional possible reasons for the discrepancy may include variations in the availability of listed medications in the study setting, the multi-centered nature of some studies, and a higher proportion of older adults with comorbid conditions in other research.

This figure was higher than in previous studies (15, 33, 34). The higher prevalence could be attributed to starting medications before confirming a specific diagnosis with objective findings (empiric treatment), the inclusion of multi-morbid patients, available medications, and varying prescribing patterns. For example, Inamdar & Kulkarni enrolled only DM patients, whereas Lim et al. included older adults regardless of disease condition. Meanwhile, this figure is considerably higher than findings from other Ethiopian hospital studies: 28.6% in northern Ethiopia (35) and 27.7% in northwest Ethiopia (12). The discrepancy may be due to differences in prescribing patterns, study settings, and study populations; for instance, the northern Ethiopia study included admitted patients across different wards. Additionally, over half of the patients on antibiotics had an infectious disease, resulting in a higher percentage of antibiotics prescribed, whereas the Beers list predominantly includes non-antibiotic drugs. The long-acting sulfonylurea glibenclamide accounted for the largest share of PIMs in both the Beers and STOPP/START criteria. Long-acting sulfonylureas, including chlorpropamide, glimepiride, and glibenclamide, can cause prolonged hypoglycemia and should therefore be avoided in older adults. Geriatric guidelines and scientific organizations recommend short-acting sulfonylureas, such as glipizide, for older adult patients to prevent prolonged hypoglycemia (18, 19). Although glibenclamide is classified as a PIM, it is included as a first-line treatment option for type 2 diabetes as an alternative to metformin in Ethiopia’s standard treatment guidelines (27). This recommendation is based on the availability and low cost of glibenclamide, making it accessible and affordable for patients.

Polypharmacy, being female, and age ≥75 were significantly associated with Beers PIMs, while age between 70 and 74 and polypharmacy were associated with STOPP/START PIMs. Consistent with other studies (11, 35–37), polypharmacy is an important predictor of PIMs. Age is another contributing factor, as supported by previous studies (37). Unlike other studies (11, 34, 35), comorbidity showed no association with the occurrence of PIMs in this study. However, earlier studies have reported an increased prevalence of PIMs in older patients with comorbidities such as diabetes, ischemic heart disease, heart failure, chronic kidney disease, cancer, osteoarthritis, osteoporosis, and anxiety (28).

This study highlights that the prevalence of polypharmacy (defined as 5 or more drugs) was 33.9%, aligning with findings from systematic reviews and meta-analyses (38–40, and). This prevalence is higher than that reported in Iran (23.1%) (41) but lower than in Germany, where polypharmacy of 5–9 drugs was reported at 58.3% and ≥10 drugs at 28.5% (40). In this study, the prevalence of potential drug–drug interactions was 47.0%. This finding differs from reports in a systematic review and meta-analysis, which showed a prevalence of 57.8% (42), in Pakistan at 70.17% (43), a multicenter study across Bern, Brussels, Cork, and Utrecht at 54% (44), and studies using Lexicomp®, Micromedex®, and DDInter checker software, which reported prevalences of 32.22, 32.93, and 22.62%, respectively (45). Scientific reasons for these discrepancies may include differences in study design (e.g., pooled prevalence in systematic reviews and meta-analyses), multicenter approaches, variations in study populations (such as admitted patients), and the use of different drug interaction checker software.

Older patients benefit from special considerations before and after drug prescriptions. To achieve this, physicians should be assisted by pharmacists and, ideally, by patients themselves (23). All health professionals should be aware of the basic changes in drug pharmacokinetics and pharmacodynamics that occur with aging. Specifically, those prescribing medications should always consider these changes to prevent compromising the health of older adult patients through inappropriate prescriptions. Pharmacists are key professionals in avoiding the use of PIMs, as they can identify possible contraindications in older adult patients for all drugs they dispense (46). In Ethiopia, current hospital reform implementation guidelines include clinical pharmacy services in inpatient, outpatient, and emergency departments. These services should be well-organized, recorded, documented, and reported (47).

An important aspect of this study is that it provides insight into the magnitude of polypharmacy, potential drug–drug interactions, and PIMs in ambulatory, chronically ill older patients in a developing setting. It encourages physicians to pay closer attention when prescribing medications, particularly for patients with cardiovascular diseases and endocrine disorders. This study has several strengths, including its prospective design, use of the latest criteria for identifying potentially inappropriate medications (PIMs), and the involvement of physicians in data collection discussions. It also emphasizes the potential for clinical pharmacists to enhance patient care by identifying, preventing, and addressing medication-related issues. However, there are limitations. First, over-the-counter medications were excluded, as only prescribed medications from patient charts were considered, potentially limiting the study’s generalizability. Second, the assessment of patients’ general health and comorbidities relied solely on available documents, which may have led to inaccurate estimates of PIMs. Finally, the study could not assess the outcomes of PIMs due to incomplete data records.

## Conclusion

This study revealed that polypharmacy, drug–drug interactions, and the prescribing of potentially inappropriate medications are common among older, chronically ill patients in Ethiopia. Polypharmacy, sex, and age were identified as contributing factors that increase the likelihood of PIM use in this population. Given the rapid growth of the older population, future studies with robust study designs are needed to further explore the adverse health outcomes and the economic burden associated with the use of PIMs.

## Data Availability

The original contributions presented in the study are included in the article/supplementary material, further inquiries can be directed to the corresponding author.
